# Biological Activity of Lactic Acid Bacteria Exopolysaccharides and Their Applications in the Food and Pharmaceutical Industries

**DOI:** 10.3390/foods13111621

**Published:** 2024-05-23

**Authors:** Shengnan Liang, Xinyu Wang, Chun Li, Libo Liu

**Affiliations:** 1 College of Food Science, Northeast Agricultural University, Harbin 150030, China; 2 Heilongjiang Green Food Science Research Institute, Harbin 150028, China

**Keywords:** lactic acid bacteria, exopolysaccharides, biosynthesis pathways, biological activity, food and medical applications

## Abstract

Exopolysaccharides are natural macromolecular bioactive substances produced by lactic acid bacteria. With their unique physiological activity and structural characteristics, they are gradually showing broad application prospects in the food and pharmaceutical industries. Exopolysaccharides have various biological functions, such as exerting antioxidant and anti-tumor activities and regulating gut microbiota. Meanwhile, as a food additive, exopolysaccharides can significantly enhance the taste and quality of food, bringing consumers a better eating experience. In the field of medicine, exopolysaccharides have been widely used as drug carriers due to their non-toxic properties and good biocompatibility. This article summarizes the biological activities of exopolysaccharides produced by lactic acid bacteria, their synthesis, and their applications in food and pharmaceutical industries, aiming to promote further research and development in this field.

## 1. Introduction

Lactic acid bacteria (LAB) are a type of Gram-positive bacteria that can survive in acidic environments and ferment carbohydrates. They include multiple genera, with *Lactobacillus* being the largest genus [1]. Previous studies have shown that LAB possess multiple physiological functions, including strengthening the intestinal mucosa, enhancing immune regulatory capacity, improving the antioxidant capacity of the body, reducing cholesterol, and resisting biofilm [2]. In addition, studies have revealed that these features of LAB are closely related to the molecules produced by their metabolism, such as bacteriocins and exopolysaccharides (EPSs). Among them, EPSs have attracted much attention due to their unique properties and functions and have become a research hotspot. The research conducted by Garcia-Castillo et al. [3] found that *Limosilactobacillus fermentum* UCO-979C has the ability to regulate the innate immune response in the stomach and affect its anti-inflammatory activity due to its production of EPS.

LAB, widely acknowledged as food-grade microorganisms for their general safety, are similarly considered harmless when it comes to the EPS they produce. The production of EPS is closely related to the growth and metabolism of LAB. Bacteria can produce capsular polysaccharides (K-antigens) (CPSs) and EPSs, wherein CPSs are covalently bound to the cell surface through phospholipid or lipid A molecules, while EPSs are released from the cell surface into the medium, forming a mucus [4]. The main focus of this paper is to introduce EPS.

EPS produced by LAB has attracted much attention due to its rich biological activities, including antioxidation [5], immune regulation [6], antibacterial action [7], hypoglycemic properties [8], intestinal microbiota regulation [9], and anti-tumor properties [10]. These characteristics make EPSs produced by LAB have broad application prospects in the fields of health care products, therapeutic drug additives, and adjuvant therapy for inflammation and cancer. In addition, EPS plays a crucial biological role in the body, protecting bacterial cells from antibiotics and toxic compounds while promoting adhesion to eukaryotic cells, thereby effectively obtaining nutrients [11]. Therefore, EPS produced by LAB has demonstrated tremendous potential in the pharmaceutical industry and is considered to have significant benefits for human health. With further research, the application value and medical significance of EPS produced by LAB will be further explored and utilized. EPS plays a significant role in the food industry, where it is widely used and exhibits multiple functional properties. Apart from its emulsifying [12], thickening, and stabilizing properties [13], EPS can also impart ideal rheological characteristics, firmness, taste, and texture to food products. For instance, it can increase the viscosity of food, reduce dehydration and shrinkage, and improve hardness [14]. It is worth noting that EPS produced in situ by LAB can reduce or eliminate the dependence on food additives, thus optimizing the food production process while also catering to modern consumers’ preference for reduced additive usage. Therefore, the application of EPS produced by LAB in the food industry not only greatly improves the quality and taste of food but also perfectly aligns with consumers’ pursuit of healthy and natural food.

The advantages exhibited by EPS in terms of activity and structure have also been applied in the pharmaceutical industry. To further explore the diversity of EPS and promote its effective application, this review delves into the biological activities of EPS produced by LAB and systematically summarizes the potential of EPS in the food and pharmaceutical industries. By systematically organizing relevant theoretical knowledge, we aim to enhance people’s understanding and application of EPS, providing strong support for its application.

## 2. Biosynthesis of EPS Produced by LAB

**Figure 1:**
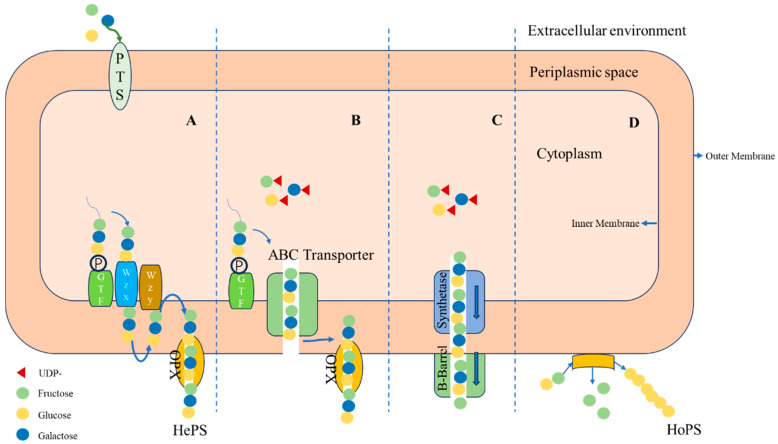
The biosynthetic pathway of EPS produced by LAB. Note: (**A**) Wzx/Wzy-dependent pathway. (**B**) ABC transporter-dependent pathway. (**C**) Synthase-dependent pathway. (**D**) Extracellular synthesis pathway.

All LAB share similar EPS biosynthesis methods [15], including extracellular synthesis and intracellular synthesis pathways (Figure 1). The extracellular synthesis pathway mainly relies on sucrase: under the action of extracellular sucrase, the precursors obtained from the cleavage of sucrose molecules are assembled extracellularly to produce EPS. The reaction formula is as follows: sucrose →β-2,6- levan + D-glucose [16]. This pathway mainly forms homo-polysaccharides, which are composed of repeated units of the same type of monosaccharide and are connected to form linear or branched structures [17]. The EPS synthesized by LAB is mainly composed of α-D-glucans with linear backbones, especially in Leuconostoc and Weissella species [18]. The intracellular synthesis pathway mainly includes the following three types: (1) The first is the Wzx/Wzy-dependent pathway [19], which is mainly involved in the synthesis of capsular polysaccharides. Firstly, the glycosyl is actively transported from the extracellular space into the intracellular space and connects with the carrier on the cell membrane to form undecaprenyl pyrophosphate (Und-P). Subsequently, the glycosyl units are linked to produce repeating units and transferred from the intracellular space to the extracellular space under the action of Wzx flippase. After modification, the polysaccharides are polymerized by Wzy protein. (2) The second is the ABC transporter-dependent pathway [20]. In this pathway, free glycosyl units bind to Und-P on the cell membrane and are continuously catalyzed to form complete polysaccharides. Subsequently, the exocytosis pump complex located on the inner membrane transports them across the inner membrane to the cell surface. (3) The third is the synthetase-dependent pathway [19]. This pathway can secrete intact polymer chains without relying on flippases. Polymerization and translocation are completed by synthetases or membrane subunits, and they are often used for the assembly of homopolymers from monosaccharide precursors. This pathway mainly synthesizes hetero-polysaccharides, which are composed of repeated units of different monosaccharides, such as pentoses (D-ribose, D-arabinose, D-xylose), hexoses (D-glucose, D-galactose, D-mannose), N-acetylated monosaccharides (N-acetylglucosamine and N-acetylgalactosamine), or uronic acids (D-glucuronic acid and D-galacturonic acid) [21,22]. Hetero-polysaccharides are mainly produced by *Lacticaseibacillus casei*, *Latilactobacillus sakei*, *Lacticaseibacillus rhamnosus*, *Lactobacillus acidophilus*, *Lactobacillus delbrueckii* subsp. *bulgaricus*, *Lactobacillus helveticus*, and *Streptococcus salivarius* subsp. *thermophilus*, among others [23].

The synthesis of EPS produced by LAB is a complex biological process that is strictly regulated by specific EPS gene clusters. These gene clusters may be located on the chromatin or plasmids of LAB [24]. Structurally, the EPS gene cluster is usually located in an operon of 11–22 kb and consists of 13–23 specific functional regions [25]. Genes related to EPS production can be divided into three categories: genes encoding the biosynthetic pathway of nucleotide sugars or other components, genes encoding glycosyltransferases, and genes required for oligosaccharide or polysaccharide processing [26]. In the EPS gene cluster of LAB, *epsA*, *epsB*, *epsC*, *epsD*, and *epsE* exhibit high conservation and stability [27]. The EPS gene clusters among different strains exhibit diversity, leading to differences in EPS characteristics. Deo et al. [28]. analyzed 106 gene clusters from 27 species of LAB. Their findings revealed that, while *epsA*, *epsB*, *epsC*, *epsD*, and *epsE* showed high conservation, *gt*, *wzx*, and *wzy* exhibited significant variability in terms of the number and composition of protein families.

## 3. The Biological Activity of EPS Produced by LAB

**Figure 2:**
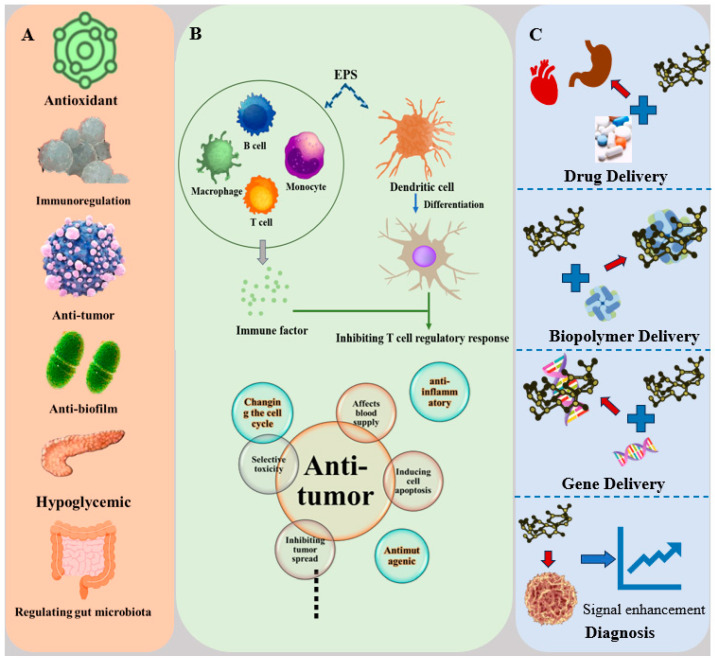
(**A**) The biological activity of EPS produced by LAB. (**B**) The mechanism of EPS regulating immune and anti-tumor activity. (**C**) The application of EPS produced by LAB in medicine.

As a unique bioactive substance, EPS produced by LAB exhibits a wide range of biological activities (Figure 2A). EPS produced by LAB can regulate the immune response of the body, increase the ability to resist pathogens, and exert antioxidant effects to protect cells from the damage caused by free radicals. In addition, its role in maintaining intestinal health and promoting microbial balance cannot be ignored. These rich biological activities make EPS produced by LAB have broad application prospects in the fields of medicine, health products, and food.

### 3.1. Antioxidant

Superoxide anion, hydroxyl radical, hydrogen peroxide radical, and nitric oxide, as single-electron reduction products of oxygen in the body, are collectively referred to as reactive oxygen species (ROS). They are ubiquitous in organisms and are inevitable products of normal cell metabolism. At appropriate concentrations, ROS are crucial for maintaining normal life activities of the body. However, when the concentration of ROS is too high, they can cause damage to biological molecules such as lipids and proteins, leading to a series of diseases including diabetes, cancer, atherosclerosis, liver disease, and chronic degenerative diseases [29]. Studies have shown that the intake of natural antioxidants can help reduce the risk of chronic diseases [30]. In recent years, EPS have garnered widespread attention for their remarkable in vitro antioxidant activity. These polysaccharides not only bolster cellular defense mechanisms but also effectively neutralize free radicals and mitigate oxidative damage triggered by ROS, safeguarding cells from harm [31].

The antioxidant capacity of EPS is closely related to elements of the environment, such as pH, temperature, oxygen content, and the types of carbon and nitrogen sources [6]. Amiri et al. [32] conducted a thorough study on the production of EPS from *Lactobacillus acidophilus* LA_5_ and *Bifidobacterium animalis* subsp. lactis BB_12_, focusing on screening the three key variables of incubation temperature, fermentation time, and yeast extract concentration. The research results indicated that these variables have a significant impact on the production of EPS. By optimizing these conditions, the yield of EPS can be effectively increased. Furthermore, the EPS produced by *L. acidophilus* LA_5_ and *B. animalis* subsp. *lactis* BB_12_ possesses potential applications as a natural antioxidant and bioactive additive. The antioxidant capacity of EPS is closely related to its structure. As Xiao et al. [33] have demonstrated, modified EPS, especially sulfated EPS, exhibits stronger antioxidant capability. Liu et al. [34]. also demonstrated that sulfated EPS exhibits stronger antioxidant capability, enhancing the activities of antioxidant-related enzymes (superoxide dismutase, catalase, and glutathione peroxidase) as well as the expression of genes (SODS, GPX2, and MT1M) in Caco-2 cells. There is controversy regarding the impact of molecular weight on the antioxidant capacity of EPS. Some scholars believe that low molecular weight promotes the exposure of hydrogen bonds in active fragments in the environment but that low molecular weight may also affect the binding of configuration-related enzymes and signals [35]. Moreover, the antioxidant capacity of EPS is also related to its monosaccharide composition. EPS rich in fucose, ribose, arabinose, and rhamnose is considered to have stronger antioxidant capability [36]. The presence of uronic acid gives EPS a negative charge, which can activate the hydrogen atoms of sugar residues through electric fields and induction, thus enhancing the ability to scavenge free radicals [37]. Yue et al. [38]. tested the antioxidant capacity of three purified components of EPS, and through correlation analysis, they also proved that the main factors affecting the antioxidant capacity of EPS are structure, molecular weight, and monosaccharide composition. EPS produced by LAB has been proven to be an effective natural antioxidant that can significantly counteract the oxidative stress caused by free radicals. Compared to synthetic antioxidants, naturally sourced antioxidants such as extracellular polysaccharides have unique advantages. Synthetic antioxidants may have potential toxicities and side effects, while natural antioxidants possess better biocompatibility and safety [39,40]. The EPS extracted from *Lactiplantibacillus plantarum* by Liu et al. [34] possesses excellent superoxide and hydroxyl radical scavenging abilities, effectively protecting Caco-2 cells from H_2_O_2_ damage and upregulating enzymes that produce antioxidants. Li et al. [41] Li et al., purified the EPS produced by *Lactobacillus helveticus* into three components, which had similar molecular weights and were composed of galactose, glucose, and mannose. The results showed that both crude EPS and purified EPS exhibited strong free radical scavenging activity, and the antioxidant capacity of crude EPS was stronger than that of purified EPS. The above research results indicate that the EPS produced by LAB exhibits significant activity in antioxidant aspects.

### 3.2. Immunoregulation

The immune response is an extremely complex and delicate process, involving precise regulation of numerous anti-inflammatory cytokines, cytokine inhibitors, and other molecules. These molecules play a crucial role in the balance and coordination of the immune system, jointly maintaining the healthy state of the body [42]. The importance of the innate immune system, as the first line of defense for the host against invasion by foreign pathogens, is self-evident. Among them, phagocytes such as macrophages, granulocytes, and monocytes play an indispensable role. They can quickly identify and phagocytize pathogens and trigger inflammation and host defense responses, thus effectively preventing the further invasion and spread of pathogens [43]. EPS regulates the immune function of the body through multiple pathways, thereby enhancing the host’s resistance to pathogens (Figure 2B). Firstly, EPS can activate immune cells such as macrophages, B lymphocytes, and T lymphocytes [44]. EPS binds to receptors on the surface of immune cells, triggering signal transduction pathways and subsequently activating the activity of immune cells. Activated immune cells can more effectively perform their functions, such as phagocytizing pathogens and secreting cytokines, thereby enhancing the immune response of the body. Monocytes are an important type of phagocyte that can phagocytize and clear pathogens and harmful substances in the body. EPS helps the body more effectively eliminate pathogens and reduce the risk of infection by enhancing the phagocytic activity of monocytes. In addition, EPS can also affect the secretion of immune factors by macrophages, such as TNF-α, IL-2, IL-6, and IL-10 [45]. Immunocytokines are an important class of signaling molecules that can regulate the activity and function of immune cells. By influencing the secretion of immunocytokines, EPS further regulates the immune response of the body. Lastly, the EPS produced by LAB promotes the differentiation of dendritic cells, facilitate their binding with immune factors, and further induces the differentiation of naive T cells into regulatory T cells, suppressing the regulatory response of T cells [6].

Activating immune cells can make them have stronger antigen recognition and killing abilities. As immune cells are activated to a higher degree, their ability to enhance their own functions and release immunomodulatory factors becomes stronger, thereby further boosting the immune capacity of the body. Zhang et al. [46] found that *L. plantarum* YW11 has several beneficial effects. It can effectively alleviate DSS-induced colitis; notably decrease the production of proinflammatory cytokines such as TNF-α, IL-1β, IL-6, IFN-γ, IL-12, and IL-18; enhance the production of the anti-inflammatory cytokine IL-10; and significantly improve intestinal microbiota. The study by Ciszek-Lenda et al. [47] delved into the impact of EPS produced by *Lacticaseibacillus rhamnosus* KL37 on inflammatory mediators in mouse peritoneal macrophages. These authors conducted a comparative analysis of the effects of EPS and lipopolysaccharides and revealed an association between EPSs and lipopolysaccharides in activating mitogen-activated protein kinases. The experimental results showed that this EPS can stimulate the production of proinflammatory cytokines and anti-inflammatory cytokines in mouse peritoneal macrophages, thus regulating inflammatory responses. Wang et al. [48] found in their research that EPS possesses significant immunomodulatory activity, which can improve the survival rate of RAW264.7 cells and enhance their phagocytic function, thereby promoting the production of NO and cytokines. Another study suggests that EPS with a higher molecular weight exhibits stronger immune activity, which can be demonstrated by the phagocytic ability of macrophages and the release of NO [49]. Wallimann et al. [50] discovered that EPS can inhibit the formation of osteoclasts from mouse bone marrow precursor cells under both normal and inflammatory conditions while promoting the deposition of mineralized matrix in osteoblasts. However, the inhibitory effect can be attenuated or blocked by TLR2 antibodies or MyD88 osteoclast precursors, suggesting that the mechanism of EPS is related to the TLR2 and MyD88 signaling pathways.

### 3.3. Antitumor Activity

Cancer is one of the main causes of high morbidity and mortality worldwide, and cancer cells are the main component of malignant tumors [51]. In response to this severe health challenge, numerous studies have focused on the anti-tumor activity of EPSs and revealed their mechanisms of action (Figure 2B). Firstly, EPSs exert anti-tumor effects by influencing the blood supply to tumors. Substances such as bacteriocins can cause ischemic necrosis in tumor cell tissue, suggesting that EPS may interfere with the blood supply to tumors through a similar mechanism, thereby inhibiting their growth [52]. Secondly, EPS can induce apoptosis in tumor cells, leading to vacuolation and cytoplasmic condensation in these cells [53]. Furthermore, EPS binds to cell receptors on the intestinal surface, enhancing the homeostasis of the intestinal mucosa and further preventing the spread of tumors. Finally, EPS can also inhibit tumor growth through a combination of various mechanisms, including altering the cell cycle of tumor cells, anti-mutagenesis, anti-oxidation, and anti-inflammatory effects [53]. It is worth noting that EPS exhibits selective toxicity and exerts little toxicity on normal cells [54].

Since Shiomi et al. [55] first reported in 1982 that the EPS produced by LAB has antitumor activity, its unique biological activity has attracted widespread academic attention. Kitazawa et al. [56] have revealed the unique anticancer role of EPS. Experimental results showed that injecting the lyophilized culture containing EPS into the abdominal cavity of tumor-bearing mice produced significant anticancer effects. This discovery strongly suggests that EPS is a key component in the anticancer action of the strain. After carboxymethylation, EPS exhibited a significant inhibitory effect on the proliferation of MCF-7 cells. This finding not only reveals a newly function of carboxymethylated EPS in cell biology but also provides a potential new strategy for cancer treatment [57]. Li et al. [58] discovered that EPS isolated from *Limositobacillus fermentum* exhibits significant anti-colon-cancer activity. By upregulating the Bax/Bcl-2 ratio, reducing mitochondrial membrane potential, and blocking the PI3K/AKT signaling pathway to arrest the cell cycle in the G1 phase, it can effectively induce apoptosis in colon cancer cells, thereby inhibiting tumor growth. Importantly, this EPS is harmless to normal human cells, demonstrating its tremendous potential as a nutritional drug for the prevention and treatment of colorectal cancer.

### 3.4. Regulating Gut Microbiota

EPS can act as a protective barrier for bacterial strains, significantly enhancing their resistance to harsh gastrointestinal environments such as those with low pH, bile salts, or various digestive enzymes, thereby increasing their survival rates in the intestine. EPS adheres to intestinal mucus in a dose-dependent manner, exhibiting a potential shielding effect that inhibits the function of specific adhesion factors on the surface of bacterial cells. It may also interfere with electrostatic interactions, thereby hindering the binding of bacteria to receptors on the mucosal surface. This process effectively weakens the adhesion ability of bacteria and impairs the recognition mechanisms necessary for the formation of stable adhesion in animal cells [59]. The research conducted by Bengoa et al. [60] indicates that the EPS produced by *Lacticaseibacillus paracasei* can be effectively metabolized by the fecal microbiota, thereby enhancing the production of beneficial metabolites such as propionic acid and butyric acid. Kuang et al. [61] successfully isolated EPS-K4 from *Bacillus amyloliquefaciens* through anion exchange chromatography, with an average molecular weight of 10067 Da. The composition of EPS-K4 was determined to be mannose, rhamnose, glucuronic acid, and glucose, with the molar ratio of each component being 40.09:23.65:11.42:17.68, respectively. Further research found that EPS-K4 exhibits significant biological activity, capable of regulating the intestinal microbiota and thereby improving symptoms in mice with colitis. Additionally, EPS-K4 effectively suppresses the levels of intestinal pathogenic bacteria, increases the number of LAB, and enhances the diversity of the intestinal microbiota. Li et al. [62] found that when the gene cluster of *Escherichia coli* EC100 is overexpressed, it generates EPS. Through further experiments, they discovered that this EPS can significantly increase the production of short-chain fatty acids and have a positive impact on the composition of intestinal microbiota. Tarique et al. [63] found that EPSs produced by *Lactobacillus delbrueckii* and *Lacticaseibacillus rhamnosus* exhibited significant biological activity within in vitro fecal fermentation experiments. These EPSs enhance the growth of certain probiotic strains to a certain extent by promoting the metabolism of carbohydrates and the production of gases and short-chain fatty acids. These studies have revealed the positive regulatory effect of EPS on intestinal microbiota and their metabolites, suggesting that EPS has the potential to become a novel prebiotic component for improving intestinal health.

### 3.5. Anti-Biofilm

Bacteria possess a unique survival strategy, which is to communicate between strains or within a strain through biofilms. This communication mechanism enables bacteria to rapidly adapt to various unfavorable environments, such as those with adverse pH values, high temperatures, metal ions, antibiotics, and other harsh conditions [17]. The formation of biofilms not only provides a protective barrier for bacteria but also promotes information transmission and resource sharing among strains. Through biofilms, bacteria can jointly respond to external challenges and maintain their ability to survive and reproduce [11]. Biofilm is a colony aggregate formed by bacteria to adapt to various adverse environments and is wrapped by extracellular polymers secreted by itself [64]. This colony aggregate is mainly formed by extracellular polymers secreted by bacteria themselves, which include EPS, proteins, and extracellular DNA. EPS plays a crucial role in the formation of biofilms [65]. During the reproduction process of bacteria, EPS produces a large amount of water-soluble substances. These water-soluble macromolecular carbohydrates are not only the key components of biofilm formation but also can interact with proteins to maintain the structure and stability of biofilms together [66]. This interaction helps biofilms maintain their integrity in complex environments, thereby protecting bacteria from external adverse factors. Surprisingly, the LAB biofilm can inhibit the growth, adhesion, surface colonization, and biofilm formation of pathogenic bacteria. The interaction between the LAB biofilm and pathogenic cells evolves in competition for nutrients and space, inhibiting the initial attachment and development of the pathogenic biofilm or reducing the growth of pathogens in established biofilms [67]. Song et al. [68] found that the EPS of *Lactiplantibacillus plantarum* can reduce the minimum elimination concentration of antibiotics on *Shigella flexneri* biofilm. Mahdhi et al. [69] found that EPS extracted from *Lactiplantibacillus plantarum* exhibits significant anti-biofilm effects. As the concentration of EPS increases, its biofilm removal ability is enhanced, and it has broad-spectrum activity against multiple biofilm-forming bacteria, such as *Staphylococcus aureus* and *Salmonella typhimurium*. Sarikaya et al. [70] demonstrated that *L. fermentum* LB-69 has a significant anti-biofilm effect against *Bacillus cereus* RSKK 863. Data analysis showed a strong correlation between the amount of mannose in EPS and anti-biofilm activity (*p* < 0.01). Therefore, these authors believe that the EPS of *L. fermentum* LB-69 has the potential dual functions of stimulating bifidobacterial growth and exerting anti-biofilm activity, making it a potential candidate for effective drugs.

### 3.6. Hypoglycemic

Type II diabetes is one of the most common chronic diseases worldwide, belonging to the category of metabolic diseases and differing from type I diabetes. Its pathogenesis is quite complex, being a chronic metabolic disorder [71]. The main characteristics of type II diabetes are insulin resistance and pancreatic cell damage, which together lead to the occurrence of chronic hyperglycemia [72]. Therefore, for the prevention and treatment of type II diabetes, it is necessary to comprehensively consider multiple factors to control blood sugar levels and reduce the occurrence of related complications [73]. Traditional diabetes treatment drugs tend to be expensive and carry significant toxicity, leading to a range of adverse reactions. Long-term use of these drugs may not only lead to a series of side effects but also cause the body to develop tolerance to the drugs, making them unsuitable for long-term dependency [74].

Research has shown that LAB exhibits significant effects in the treatment and prevention of diabetes [75]. LAB can regulate the structure of the intestinal microbiota, reduce inflammatory responses, and thereby alleviate insulin resistance. At the same time, they can also regulate the release of gastrointestinal hormones, effectively controlling the absorption and balance of energy. Even more noteworthy is that LAB can regulate the expression of related genes in sugar metabolism pathways, helping to maintain blood glucose homeostasis and effectively improving the symptoms of type II diabetes. This discovery provides new ideas and methods for the treatment of diabetes. Zhang et al. [76] conducted an experiment involving three probiotics: *Lacticaseibacillus paracasei* 1F-20, *L. fermentum* F40-4, and *Bifidobacterium animalis* subsp 01. The experiment found that these probiotics survive in the gastrointestinal tract, upregulating the expression of peptide YY and glucagon genes, thereby reducing lipid accumulation and promoting glucose absorption. Notably, *B. animalis* subsp 01 effectively regulates sugar metabolism in the livers of diabetic rats, lowering hyperglycemia. As a derivative of probiotics, EPS produced by LAB has demonstrated significant effects in hypoglycemia. Huang et al. [77] conducted a thorough analysis of the EPS secreted by *L. plantarum* H31 isolated from kimchi. The results showed that EPS is primarily composed of mannose, which has the ability to inhibit the activity of various digestive enzymes and plays a crucial role in improving hyperglycemic symptoms. This discovery reveals the mechanism by which the EPS secreted by *L. plantarum* reduces blood glucose concentration by inhibiting the activity of relevant enzymes in the body, making it an important probiotic metabolite for alleviating type II diabetes. This research finding provides a beneficial exploration direction for the development of novel and efficient diabetes treatment methods.

## 4. Application of EPS Produced by LAB

### 4.1. The Application of EPS Produced by LAB in the Food Industry

EPS produced by LAB, as a natural biological macromolecule, has gradually attracted widespread attention in the field of food applications. With its unique physicochemical properties, such as good water solubility, stability, and distinctive taste and viscosity, EPS produced by LAB provides ample innovative opportunities for the food industry. EPS produced by LAB can play a significant role in various fields, including dairy products, beverages, and baked goods, serving as a thickening agent, stabilizer, or taste modifier to significantly enhance the quality and taste of food (Table 1).

#### 4.1.1. Fermented Dairy Products

As a natural stabilizer, thickener, and gelling agent, EPS produced by LAB plays an indispensable role in fermented dairy products. Its unique properties can effectively change the rheological and emulsifying properties of fermented milk, giving it a unique and pleasant texture and full-bodied taste. Moreover, it can significantly enhance the viscosity of fermented dairy products, effectively reducing the separation of whey, thereby greatly improving the overall quality of fermented milk. The addition of this natural ingredient not only enriches the taste layers of dairy products but also enhances their nutritional value and health attributes, providing consumers with a more high-quality and healthy dairy product choice [92]. During the production process, EPS is produced slowly and stably through the metabolic action of LAB as the fermentation process advances. At the same time, EPS interacts synergistically with casein coagulation during the gel formation process, working together to exert its effects. With the gradual accumulation of EPS and the formation of a network structure, the three-dimensional network structure of casein is constructed uniformly and finely, which significantly improves the viscosity and texture of the fermented milk. This process makes the taste of fermented milk smoother and finer and makes the texture more uniform and stable [82,93,94]. Zhang et al. [80] found that yogurt fermented by LAB capable of producing high-molecular-weight EPS has a superior gel state. This advantage may be attributed to the tight connection between EPS and proteins, which effectively fills the network structure of casein clusters, thereby forming a more compact and stable gel network structure. In addition, during the production of yogurt, issues such as low viscosity, easy breakage of curds, and significant whey separation often arise. These problems not only compromise the taste of yogurt but also severely affect its quality. Fortunately, the EPS-producing LAB provide an effective approach to addressing these issues [95]. The viscosity of yogurt containing EPS increases significantly, and the viscosity of EPS is mainly influenced by its molecular weight, the linearity and stiffness of the main chain, and the complexity of the side chains [96]. In addition, the interaction between EPS and other components in the system is also a crucial factor determining its viscosity [97]. Specifically, EPS with negative charges exhibits higher viscoelasticity, stiffness, and viscosity due to its unique physicochemical properties [98]. These properties, along with the production time of EPS during fermentation, jointly determine the distribution pattern of EPS in the microstructure, which further affects its interference level in the gelation process and protein matrix. The situation becomes even more complex when EPSs interact with proteins. At the isoelectric point of proteins, they are in a neutral-charge state. If EPS can interact with the protein network through electrostatic interactions, it may interfere with the coagulation process of proteins, leading to the formation of a continuous branched protein network, which further enhances the viscoelasticity of the system [97]. However, if there are surface inert characteristics between EPS and proteins (such as casein), such as neutral or positively charged EPS, depletion effects or phase separation may occur, leading to the formation of protein and EPS aggregates and reducing viscoelasticity [99]. Yogurt containing EPS undergoes lower levels of mechanical damage during pumping, stirring, and filling processes, and its curds have a certain resistance to heat treatment and physical stress. Therefore, there is no need to add additional stabilizers when producing yogurt. This production process not only simplifies the workflow but also improves the quality and taste of the product.

#### 4.1.2. Plant-Based Dairy Products

With the development of society, people have gradually focused their attention on vegetarian and green food, so plant-based yogurt has become a hot topic in today’s society. Therefore, the production and consumption of plant-based yogurt have begun to increase significantly, but the defects in flavor and taste of plant-based yogurt are still issues that need to be addressed. The starter cultures for plant-based yogurt products are usually LAB, *yeast*, and *Acetobacter aceti*, and this process is similar to the fermentation process of cereal products [100]. When LAB are used as starter cultures, their ability to produce EPS plays a crucial role in plant-based yogurt, as the EPS formed can improve its taste and texture characteristics. Therefore, manufacturers are highly interested in screening and developing LAB that produce EPS, aiming to replicate the taste and quality of dairy products. Both single LAB starter cultures and mixed LAB starter cultures improve the texture of plant-based products by producing EPS [101,102]. Previous studies have found that EPS produced by *Lactiplantibacillus plantarum* Lp90 can improve the rheological properties of oat-based foods, but the improvement effect disappears as the storage time of the product extends [103]. Lorusso et al. [104] found that EPS synthesized by *W. confusa* DSM 20194 improved the viscosity and water-holding capacity of quinoa-fermented beverages by generating a dense network structure with quinoa protein, thereby enhancing the acceptability of the beverage. Similarly, Zannini et al. [83] also found a similar phenomenon. The EPS produced by *W. cibaria* MG1 can improve the water-holding capacity and viscosity of quinoa-based yogurt drinks by forming a dense network structure with quinoa protein. This is mainly attributed to the network structure formed by EPS and protein. Therefore, it is speculated that EPS produced by LAB may affect the quality of plant-based foods by altering their protein conformations. Considering the above factors, the use of EPS-producing LAB to ferment plant-based yogurt is a promising approach that can replace the addition of additives to plant-based yogurt and also meet the market demand for fewer additives.

#### 4.1.3. Cheese

EPS can form stable gels and prevent water from leaking out of the pores in cheese, which is the main reason for its soft texture. The interaction between protein and water further enhances the viscoelasticity of cheese [105]. Due to its excellent water-holding capacity, EPS produced by LAB can significantly enhance the viscosity and optimize the texture of fermented milk. During the production and fermentation process of low-fat and non-fat cheese, this characteristic makes it an ideal natural stabilizer, eliminating the need for additional chemical stabilizers and ensuring the naturalness and safety of the product [106,107]. Research has shown that the addition of EPS to low-fat mozzarella cheese results in a higher water content and better melting properties compared to control cheese. This positive effect is mainly attributed to the accumulation of EPS in the cheese whey, which can reduce the concentration of whey protein and affect drying efficiency without adversely affecting the viscosity of the whey [108]. Surber et al. [109] found that the use of EPS-producing *Lactococcus lactis* in cheese making will give cheese a higher hardness, and the texture and dehydration-induced syneresis of cheese depend on the type of EPS and shear strength. Reyes et al. [110] conducted a thorough study on the impact of phospholipase A1 (PL-A1) and EPS-producing bacteria on cheese yield, microstructure, and texture. The results showed that while EPS could significantly increase the moisture content and fat retention rate in cheese, thus boosting the yield, it did have an impact on the overall flavor of the cheese. This indicated that although EPS could increase cheese yield, it also came with the sacrifice of some of the cheese’s fine flavor. However, the experimental data revealed that when PL-A1 was used in combination with EPS, the cheese produced exhibited optimal performance in both flavor and texture. This discovery provides new ideas and strategies for optimizing cheese production processes and enhancing the overall quality of the product.

#### 4.1.4. Bakery Products

The properties of EPS produced by LAB are similar to those of water colloids in bread improvers, and it can effectively reduce the reliance on additives in the dough during bread making [23]. The addition of EPS to the dough can not only significantly enhance the water absorption capacity of wheat dough but also improve the strength of gluten, making the dough more extensible and positively affecting its gas-holding properties. Especially in the making gluten-free products or bean dough with low protein content or a lack of gluten structure, the addition of EPS has become particularly important. By adding EPS, high-quality gluten-free products can be produced that have a smoother taste, slower aging speed, longer shelf life, and a viscoelasticity that is acceptable to a wide range of consumers [111]. The improvement mechanism mainly lies in the formation of a network structure through the arrangement of hydrogen bonds among the linear macromolecular glucans in EPS, which enhances the stability and gas-holding properties of the dough [112]. The dryness and hardness of bread are mainly caused by the degradation of starch and the loss of moisture. EPS can slow down the aging process by binding water and preventing water loss, thereby delaying starch crystallization [113]. *Lactobacillus sanfranciscensis* has the ability to produce EPS, which can effectively enhance the viscosity and gas-holding properties of bread dough when applied in bread making. Through this effect, the hardness of the bread crumb is reduced and the volume of the bread is increased, thereby improving the overall quality of the bread [114]. Sourdough rich in polysaccharides can also significantly improve the quality of bread, making it have a more fluffy structure, better elasticity, and higher water retention [115].

#### 4.1.5. Meat Products

Since ancient times, LAB has played an important role in the processing and production of meat products in the evolution of human civilization, creating a wide range of traditional foods around the world [116]. LAB can not only reduce the content of nitrite in meat products but also significantly enhance their antioxidant properties and effectively inhibit the growth of spoilage bacteria and pathogenic bacteria, thereby extending the shelf life of the products [117,118]. Moreover, LAB can produce EPS, and when the production of EPS reaches a certain level, it can further improve the quality and structure of meat products, especially for the improvement of low-fat meat products [119]. Therefore, the use of LAB that produces EPS can not only replace commonly used additives in meat products (such as hydrophilic colloids and phosphates) but also meet the modern consumers’ demand for foods with fewer additives. In meat processing, LAB are often used in fermented raw sausages and cured ham. The main role of LAB in these products is to improve the taste and texture of the products, as well as extend the shelf life of meat products. These effects are achieved through the production of EPS, and the most commonly used LAB are *Lacticaseibacillus* and *Pediococcus* [119]. According to the research by Hilbig et al. [120], the use of EPS-producing *L. sakei* TMW 1.411 or *L. curvatus* TMW 1.1928 can produce a spreadable fermented raw sausage with a lower fat content. This product is significantly softer and easier to spread compared to the control samples that do not produce EPS. Dertli et al. [121] found that *Lactiplantibacillus plantarum* and *Leuconostoc mesenterides* subsp. *mesenteroides* that produce EPS produce sausages that are more resilient compared to those fermented by LAB that do not produce EPS, meeting the texture and quality demands of consumers. Therefore, LAB that can produce EPS are a very promising method for improving the texture of meat products. However, there are still many shortcomings in the widespread use of EPS-producing LAB in the meat products industry. Currently, only a few EPS-producing LAB are applied in meat processing, and the impact of most EPS-producing LAB on specific parameters of meat products still needs further exploration. With the ongoing research on the impact of EPS produced by LAB on meat products, LAB capable of producing EPS will be more widely utilized in meat processing and gradually supersede traditional additives, thereby providing consumers with a broader array of healthier and more delicious food options.

### 4.2. Application of EPS Produced by LAB in the Pharmaceutical Industry

#### 4.2.1. Drug Delivery

EPS produced by LAB is not only easy to prepare and low cost but also environmentally friendly and biodegradable. Its rich content of hydroxyl, carboxyl, and amino groups makes it a quality material for preparing hydrogels [122]. These properties enable it to effectively capture biomolecules (such as drugs) within its internal structure or adsorb them to its outer surface. This capturing and adsorbing effect not only helps to prolong the residence time of drugs in the body, thereby improving the bioavailability of drugs, but also contributes to reducing the required drug dosage and thus decreasing the cytotoxicity of drugs [123]. Therefore, EPS has broad application prospects in the field of drug carriers. EPS serves as an ideal carrier for drugs, primarily due to the following reasons: Firstly, EPS exhibits excellent biocompatibility and safety, which means it can minimize stimulation and immune response to human tissues, ensuring the safety and reliability of the drug delivery process [124]. Secondly, the structural characteristics of EPS endow it with abundant functional groups and modifiable side chains, which provide the possibility for chemical or biological modifications. This can further improve the stability and permeability of the carrier and optimize the drug encapsulation effect [125]. In addition, EPS also possesses the ability to bind to specific receptors, which enables targeted drug delivery, significantly increasing the local concentration of drugs at the target site and enhancing the therapeutic effect [126]. In addition to the above advantages, EPS also has characteristics such as renewability, sustainability, biodegradability, and affordability [127]. These features make EPS an economical and environmentally friendly drug delivery carrier. Pradeepa et al. [128] used *Lactiplantibacillus plantarum* EPS to prepare gold nanoparticles and found that the nanostructures exhibited superior antibacterial activity compared to free drugs. This activity was mainly mediated through penetration, loss of cytoplasmic contents, and cell lysis. Kalimuthu et al. [129] used bacterial EPS as a carrier for quercetin to achieve targeted release to breast cancer cells, which exerted concentration-dependent toxicity on breast cancer cells and even enhanced the effect of quercetin.

#### 4.2.2. Biopolymer Delivery

Attention has been focused on vaccines, proteins, peptides, hormones, and small interfering RNA (siRNA) for the treatment of fatal diseases, and the frequency of using such treatments has been increasing in recent years. However, the key lies in ensuring biological macromolecules cross membranes unhindered, as they are readily eliminated by the liver or other tissues during body transmission, hindering their arrival to the target site. For example, amylopectin nanoparticles carry cholesterol protect insulin from enzymatic degradation [123]. In addition, in the research on oral insulin delivery, the use of insulin-loaded dextran sulfate and chitosan nanoparticles has revealed that these nanoparticles exhibit good protective effects in simulated intestinal fluid [130]. In the field of vaccination, EPS can be used as an antigen carrier or antigen itself in the preparation of vaccines, stimulating the formation of antibodies and providing strong immune effects against several microorganisms [131]. Furthermore, when conjugated with proteins, purified capsular polysaccharides of *Haemophilus influenzae* type b can be used as a component of multiagent vaccines against various infections (such as meningitis) among children [132]. Therefore, EPS has significant potential value in the production of recombinant macromolecular biopharmaceuticals.

#### 4.2.3. Gene Delivery

Polymers, especially polymers from microorganisms, are conducive to transporting genetic materials, so EPS has been expanded in the field of gene delivery. Raemdonck et al. [133] found that cationic dextran-based nanocarriers combined with photosensitizers help deliver siRNA across the plasma membrane into the cytosol and achieve transfection in liver cancer Huh-7 cells. Han et al. [134]. also found that nanoparticles formed by the complexation of water-soluble 6-amino-curdlan with siRNA can effectively deliver siRNA to human cancer cells and mouse primary cells and reduce the target mRNA level by 70–90%. These studies have demonstrated that EPS has the role of a non-viral vector for gene delivery, which can be used in the treatment of cancer genes, but specific applications still require further research and clinical trial development.

#### 4.2.4. Diagnosis

EPS-based nanocarriers have been used in various medical diagnostic and bioimaging applications. Studies have shown that Fe_2_O_3_-dextran cross-linked aggregates can be used for in vivo molecular diagnosis, such as fluorescence molecular tomography, positron emission tomography, magnetic resonance imaging, and diagnostic magnetic resonance [135,136]. Amino-modified cholesterol branched proteins can enhance the fluorescence intensity of tumor cells, improve the sensitivity of imaging, and promote the development of medical imaging [137]. Biopolymers can be grafted with specific types of structures. Compared with traditional complexes that require grafting, cholesterol-grafted pullulan nanocomposites exhibit high autofluorescence in cancer cells [138].

In summary, EPS’s unique functional properties and biological activities suggest vast potential in drug delivery and medical diagnosis despite the slow research progress and its unclear in vivo bioavailability (Figure 2C). Furthermore, the potential applications of EPS and its derivatives in emerging therapeutic areas, particularly in diabetes, cancer treatment, and vaccination, still warrant in-depth exploration and research globally.

## 5. Conclusions

Due to its safety, non-toxicity, and unique biochemical properties, EPS produced by LAB has been widely used in the food and pharmaceutical industries. As a potential prebiotic, EPS produced by LAB not only broadens its application scope but also provides more food-derived materials for the development of prebiotics. The addition of EPS to functional foods and medical health products is also an effective means to enhancing the added value of the products. The unique structure and properties of EPS lay the foundation for its applications in industrial fields such as biomaterials, wastewater treatment, and cosmetics. However, the process of EPS synthesis through natural strain fermentation is complex and costly, and its production is still in the laboratory stage, limiting its large-scale industrial production and application. The current research is primarily concentrated on boosting the production of EPS by optimizing fermentation conditions and media composition and by employing genetic engineering techniques. Currently, only a few bacterial EPSs are being used for commercial purposes. With the continuous development of biotechnologies such as genetic engineering and metabolic engineering, the production efficiency and quality of EPS will be further improved. This will reduce production costs and promote the widespread application of EPS in industrial and commercial fields. At the same time, the development of biotechnology will also provide more possibilities for studying the structure–activity relationship and metabolic pathways of EPS, further exploring its potential value. Due to the complexity of EPS’s structure–activity relationship and metabolic pathways, its mechanism of action in food, medicine, and other fields still needs further exploration to fully realize its potential value.

## Figures and Tables

**Table 1:** Application of EPS produced by LAB in food.

Strain	Monosaccharide Composition	Application	Reference
*Lactiplantibacillus plantarum* B51	-	Shorten the debittering time of olives and inhibit cell apoptosis	[78]
*Lactiplantibacillus paraplantarum* KM1	Glucose, galactose, and mannose	Emulsifier	[12]
*Streptococcus salivarius* subsp. *thermophilus*	Glucose, galactose, and rhamnose	Increase viscosity	[79]
*Lacticaseibacillus rhamnosus* LH43	Mannose, rhamnose, galacturonic acid, glucose, and galactose	Increase yogurt viscosity and improve texture	[80]
*Lactiplantibacillus plantarum*	-	Improve the rheological properties of plant-based milk	[81]
*Lacticaseibacillus rhamnosus* LH18	Mannose, rhamnose, galacturonic acid, glucose, and galactose	Improve yogurt gel properties	[82]
*Weissella cibaria* MG1	-	Improve the water-holding capacity of quinoa milk	[83]
*Lactobacillus buchneri* FUA3154	Glucose, galactose, and rhamnose	Affect the rheological properties of sorghum dough	[84]
*Streptococcus salivarius* subsp. *thermophilus*	-	Increase the flavor and chewiness of cheese	[85]
*Leuconostoc mesenteroides* XR1	Glucose and galactose	Improve the flocculation and thermal stability of yogurt	[86]
*Lactobacillus helveticus* MB2-1	Mannose, rhamnose, glucuronic acid, glucose, galactose, arabinose, and fucose	Improve the viscosity, texture, and microstructure of fermented milk	[87]
*Streptococcus salivarius* subsp. *Thermophilus* ZJUIDS-2-01	Glucose, galactose, N-acetyl-D-galactosamine, and rhamnose	Improve the emulsification and flocculation characteristics	[88]
*Lactiplantibacillus plantarum* MC5	-	Increase apparent viscosity and elastic modulus, reduce dehydration shrinkage	[89]
*Lactiplantibacillus plantarum* RS20D	Glucose, galactose, and glucosamine	Improve texture and reduce dehydration shrinkage	[90]
*Levilactobacillus brevis* UCLM-Lb47, *Leuconostoc mesenterides* subsp. *mesenteroides* 6F6-12 and *Leuconostoc mesenterides* subsp. *mesenteroides* 2F6-9	-	High water-holding capacity and oral viscosity value, improving texture	[91]
